# Priming adult stem cells by hypoxic pretreatments for applications in regenerative medicine

**DOI:** 10.1186/1423-0127-20-63

**Published:** 2013-08-29

**Authors:** Claudio Muscari, Emanuele Giordano, Francesca Bonafè, Marco Govoni, Alice Pasini, Carlo Guarnieri

**Affiliations:** 1 Department of Biomedical and Neuromotor Sciences (DIBINEM), University of Bologna, Via Irnerio 48, 40126, Bologna, Italy; 2 National Institute for Cardiovascular Research, Bologna, Italy; 3 Laboratory of Cellular and Molecular Engineering “Silvio Cavalcanti,” Department of Electrical, Electronic, and Information Engineering “G. Marconi” (DEI), University of Bologna, Cesena, FC, Italy; 4 BioEngLab, Health Sciences and Technologies-Interdepartmental Center for Industrial Research (HST-CIRI), University of Bologna, Ozzano Emilia, BO, Italy

**Keywords:** Hypoxia, Reoxygenation, Preconditioning, Stem cell, Apoptosis, Cell differentiation

## Abstract

The efficiency of regenerative medicine can be ameliorated by improving the biological performances of stem cells before their transplantation. Several ex-vivo protocols of non-damaging cell hypoxia have been demonstrated to significantly increase survival, proliferation and post-engraftment differentiation potential of stem cells. The best results for priming cultured stem cells against a following, otherwise lethal, ischemic stress have been obtained with brief intermittent episodes of hypoxia, or anoxia, and reoxygenation in accordance with the extraordinary protection afforded by the conventional maneuver of ischemic preconditioning in severely ischemic organs. These protocols of hypoxic preconditioning can be rather easily reproduced in a laboratory; however, more suitable pharmacological interventions inducing stem cell responses similar to those activated in hypoxia are considered among the most promising solutions for future applications in cell therapy. Here we want to offer an up-to-date review of the molecular mechanisms translating hypoxia into beneficial events for regenerative medicine. To this aim the involvement of epigenetic modifications, microRNAs, and oxidative stress, mainly activated by hypoxia inducible factors, will be discussed. Stem cell adaptation to their natural hypoxic microenvironments (niche) in healthy and neoplastic tissues will be also considered.

## Review

### Introduction

Stem cells (SCs) are currently evaluated as a tool to repair the irreversible tissue damages that permanently impair organ function. In the last decade, the increasing knowledge of SC biology has widely encouraged preclinical and clinical studies of regenerative medicine. SCs are unspecialized cells maintaining their proliferative and differentiative capacity throughout the life of an individual [1]. Their ability to divide and remain in the undifferentiated state, i.e. the self-renewal process, is a specific characteristic of these cells that can be accomplished by two mechanisms known as obligatory asymmetric replication and stochastic differentiation [2]. The former splits SCs into two daughter cells, one of which retains the property of self-renewal while the other begins to differentiate; the second requires the participation of SCs that generate two identical daughter cells together with SCs that generate only differentiating cells.

According to their differentiation potential, or potency, mammal SCs are classified as [3]: totipotent, namely the zygote and its first daughter cells which give rise to the entire organism; pluripotent, i.e. the blastocystic cells that differentiate into any of the three germ layers; multipotent, that are present in the fetus and born organisms and can differentiate into multiple, but limited cell types; oligopotent, such as the lymphoid or myeloid SCs, that differentiate into a few cell types; unipotent, that produce only one cell type but are still self-renewing. Similarly to unipotent SCs, progenitor cells are also unipotent but can divide only a limited number of times.

Furthermore, SCs can be distinguished into two categories: pluripotent embryonic SCs (ESCs) and multipotent or unipotent adult (somatic) SCs, the latter being present in near all post-natal tissues including bone marrow, blood, adipose tissue, skin, liver, muscle, and brain [4]. The picture is completed by the so-called induced pluripotent SCs (iPSCs), that are not naturally occurring SCs but are produced using virtually all types of cells by forcing the expression of a few genes that provide pluripotency [5].

An open question concerns the putative property of adult SCs to differentiate into cell phenotypes independent from the expected commitment by the tissue where they reside [6]. This plasticity of adult SCs might contribute a certain advantage for in-vivo tissue repair. For instance, it has been suggested that after an organ ischemic insult bone-marrow mesenchymal SCs (BM-MSCs) are mobilized into blood and recruited in the injured tissue where they could in part participate to the regeneration process via transdifferentiation [7]. Therefore, the interest on adult SCs, as opposed to ESCs, has rapidly increased because the former are easily accessible in the patient, do not rise ethical concerns, retain a reduced risk of tumor formation, do not require immunosuppressive treatments to prevent rejection, and could show some plasticity. However, although multipotent SCs show many characteristics that can be suitable for clinical applications, their use in regenerative medicine is often hampered by their poor survival after the engraftment [8-10]. Indeed, the injured tissue is usually fibrotic and poorly perfused; hence, the insufficient availability of oxygen and nutrients renders both grafted and resident SCs more susceptible to lethal damage.

Strategies have been proposed to ameliorate the repair of post-ischemic tissues, including SC-induced neovascolarization [11-13] and SC preconditionings able to improve SC survival after transplantation [14,15]. Both approaches are considered hot topics of the research in the field since they could significantly improve the clinical outcome of cell therapy.

**Figure 1:**
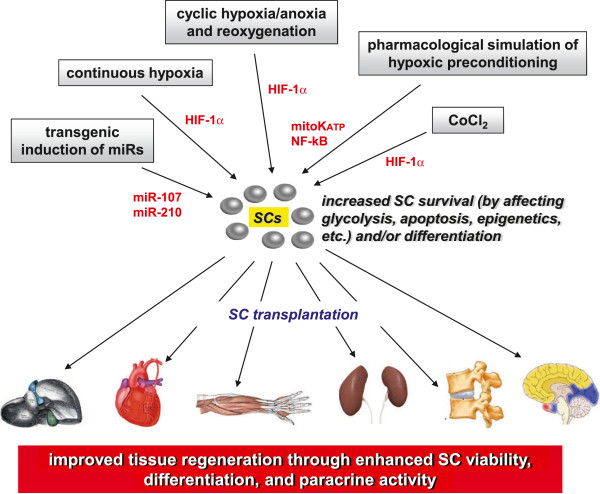
**Efficacy of hypoxic pretreatments of adult SCs in regenerative medicine.** Grafted SCs become more resistant to the death stimuli that are present in the injured tissues through ex-vivo hypoxic pretreatments (continuous hypoxia or cyclic hypoxia-anoxia and reoxygeneation) or by administration of CoCl_2_. Pharmacological agents exerting intracellular key effects of hypoxic preconditioning, such as diazoxide, can also be effective. The most common sensor activated under low oxygen tensions is HIF-1α which increases cell survival by stimulating several pathways, including glycolytic flow, Akt phosphorylation, and miRs upregulation. Transgenic induction of miR-107 and miR-210, which are mainly expressed after hypoxia, also provides protection to SCs against the engraftment injury.

Among the experimental procedures employed in the last decades for attenuating the damage induced by acute and severe ischemic events, in-vivo organ pretreatments with brief cycles of ischemia and reperfusion, namely “Ischemic Preconditioning” (IP), has been widely recognized as the best protective maneuver [16,17]. Nowadays, these basic experiences have been successfully translated into clinical interventions to prevent the potential damage due to the temporary interruption of blood perfusion during surgery [18-20]. In a similar manner, hypoxia-dependent pretreatments on SCs have been investigated to increase their survival for applications in regenerative medicine (Figure 1). Here, we will consider at first the effects exerted by hypoxia on SC viability in their natural microenvironment, to underline what molecular and cellular adaptations they develop to face this unusual condition. Then, suitable methods inducing SC protection after exposure to different protocols of low oxygen tension will be discussed, as well as the need to discover pharmacological treatments triggering the same intracellular signaling pathways leading to hypoxic adaptation. Finally, the role of non-damaging hypoxic conditions in enhancing SC proliferation and differentiation will be also described.

### Lesson from SCs resident in a natural hypoxic microenvironment

#### Basic features of SC niche

The fate of SCs in their natural environment is regulated by intrinsic and extrinsic signals balancing both cell self-renewal and differentiation. The extrinsic factors are included in the term “niche” that was first proposed in 1978 by Schofield, who defined a somehow specific microenvironment supporting the cells [21]. In their niche, SCs are supposed to be influenced by different molecules such as cytokines and growth factors (e.g. basic fibroblast growth factor (bFGF), bone morphogenetic proteins (BMPs), stem cell derived factor 1 (SDF-1), stem cell factor (SCF), leukemia inhibiting factor (LIF), Wnt/β-catenin [22]), extracellular matrix (ECM) molecules (e.g. hyaluronan [23]), some differentiated cells (e.g. fibroblasts, endothelial cells), and low O_2_ concentrations [24]. Niches of mammalian adult SCs have been described in various tissues and hypoxia appears to represent a stimulus promoting self-renewal [25]. For instance, two distinct niches for hematopoietic stem cells (HSCs) have been identified in bone marrow: the hypoxic endosteal niche and the vascular niche [26]. While the former is hypothesized to maintain SCs in a quiescent and undifferentiated state through the hypoxic environment, the latter should promote both SC proliferation and differentiation. In this regard, Notch signaling seems to be critical since its inhibition reverse the effects of hypoxia on the maintenance of stemness [27].

In the attempt to explain why hypoxia is needed for the maintenance of a SC pool, some studies suggested that mild-low levels of O_2_ can minimize the damages caused by oxidation, especially towards DNA [28]. Indeed, according to the clonogenic theory, no changes in gene structure must occur in dividing SCs that have to be the exact copy of their mother cells. However, this concept is under debate since hypoxia has also been reported as increasing, rather than blunting, oxidative stress [29].

#### Role of hypoxia inducible factor-1α in SC adaptation to hypoxia

One of the most powerful way followed by SCs to adapt to the low oxygen tension that characterizes the niche is the production of high levels of hypoxia inducible factors (HIFs) [30]. HIFs are activated when oxygen tension falls below 5% and their concentrations increase in parallel with decreasing oxygenation [31]. HIFs are transcriptional factors essential for cell responses and adaptation to hypoxia whose active form results from the interaction of α and β subunits; the former includes HIF-1α, HIF-2α, and HIF-3α, the latter HIF-1β and HIF-2β. Mechanisms of activation and target genes have been better documented for the HIF-1α subunit [32]. Under normoxic conditions, the prolyl residues in the HIF-1α oxygen-dependent degradation domain (ODD) are hydroxylated by at least three different HIF prolyl hydroxylases (PHDs) [33]. Besides molecular oxygen, hydroxylation needs the presence of 2-oxoglutarate and reduced iron ions. Moreover, a factor inhibiting HIF (FIH) hydroxylates a specific asparaginyl residue which prevents the following recruitments of co-activator p300/CBP in the consensus sequences. The hydroxylated ODD is then recognized by von Hippel-Lindau protein (VHL), an E3 ubiquitin ligase. When oxygen tension is low, PHDs are not hydroxylated, hence they become inactive and HIF-1α is stabilized because it is not degraded by the proteasome system [32]. Besides hypoxia, certain intracellular metabolites, including reactive oxygen species (ROS), fumarate, succinate, and potentially 2-hydroxyglutarate, inhibit PHD and FIH activities, resulting in HIF-1α stabilization [34]. HIF-1α thus heterodimerizes with HIF-1β, also known as arylhydrocarbon receptor nuclear factor (ARNT), and translocates into the nucleus where it binds to hypoxia response elements (HRE) along with the co-factors E1A binding protein p300 (EP300), jun proto-oncogene (c-JUN), and cAMP responsive element binding protein (CREB) [35]. This leads the modulation of up to 200 genes involved in several processes including angiogenesis, glycolysis, mitochondrial respiration and biogenesis, production of erythropoietin, redox homeostasis, cell proliferation and cell apoptosis, in both normal and tumor cells [36]. More recently, several other modulators of HIFs stability and gene transcription have been discovered, such as chaperonins, transcriptional co-factors, sirtuins, ascorbate, nitric oxides, microRNAs, oncogenes, tumor suppressors, and inflammation factors [34,37].

#### Epigenetic changes induced by hypoxia

**Figure 2:**
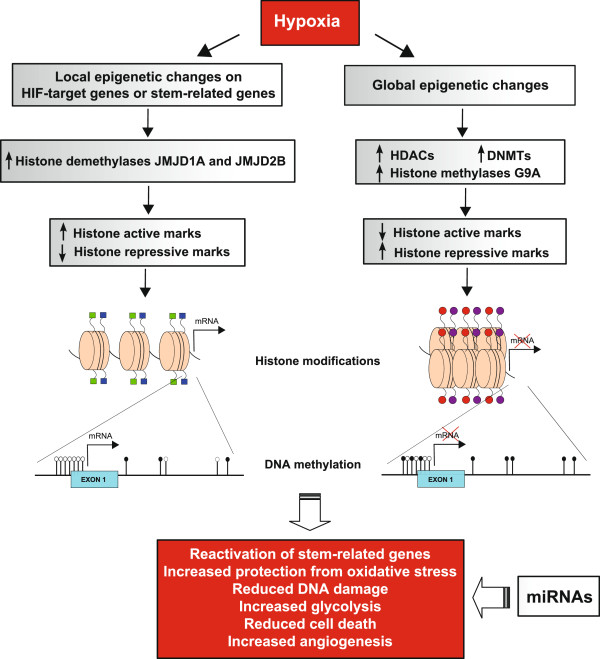
**Schematic representation of hypoxia-induced epigenetic changes.** Hypoxic conditions modulate SCs expression profile via several mechanisms, included epigenetic modifications. The induced upregulation of HIF-1α and HIF-2α, drives the activation of several target genes. Also stem-related genes, such as *OCT-4*, could be re-expressed. The chromatin configuration of their promoter region becomes accessible to transcription factors, also consequently to the upregulation of histone demethylases JMJD1A and JMJD2B, that catalyse the removal of repressive histone marks (H3K9me2/3). Histone tails are characterized by active histone modifications (light or dark blue squares), such as acetylated H3-H4 or H3K4me2/3, and DNA is unmethylated (white rounds) at the promoter CpG sites (black lines). Hypoxia also induced global repression of gene transcription, associated with the upregulation of chromatin modifier enzymes, such as histone deacetylases (HDACs) and demethylases (G9a), that drive the formation of histone repressive marks (red and purple rounds), such as deacetylated H3-H4, H3K9me2/3 or H3K27me3. DNA methylation global level increases consequently to the upregulation of DNA methyltransferases (DNMTs) and gene expression is silenced. MiRNAs additional control of transcription and translation contributes to generate a gene expression profile that allows to reactivated stem-related genes, increase protection from oxidative stress, reduce DNA damage, increase glycolysis and angiogenesis, with the final result of enhancing cell viability and their regenerative potential.

The changes in gene expression upon hypoxic stress are tightly associated with modifications in chromatin structure by histone modifying and chromatin remodeling complexes, that are referred to as epigenetic regulation of transcriptional activity (Figure 2) [38]. In this regard, it has been reported that during hypoxia, the SWI/SNF chromatin remodeling complex [39] is recruited to the promoter of the HIF-1α gene, where it is required for expression of HIF-1α mRNA [40] as a conserved feature in animal evolution [41].

HRE-containing gene promoters are affected by upregulated HIF-1α together with its co-activators involved in the epigenetic regulation of histone marks and DNA methylation. The former includes the acetylation and/or methylation of specific residues of H3 and H4 histone tails, the latter the adduction of methyl group on cytosine preceding guanine (CpG) in the DNA sequence of the target gene promoter. Indeed, the interplay between these chemical modifications and the protein complexes influencing chromatin architecture leads to the typical hypoxic transcriptional configuration, where HIF target genes are upregulated although general transcription in the cell is substantially inhibited [42].

Histone methylases and demethylases play an important role as enzymes involved in this epigenetic control. The impact of histone methylation appears context-dependent: tri-methylation of lysine 27 in H3 histone tail (H3K27me3) indicates transcriptionally repressed chromatin. The same holds true for methylated H3K9. On the other hand, the promoter region of active genes appears enriched in methylated H3K4 and/or H3K36 [43].

Interestingly, the Jumonji domain containing dioxygenases (JMID) group of histone demethylases (JHDMs), and in particular JMJD1A and JMJD2B, are induced under hypoxic conditions by the overexpression of HIF-1α and HIF-2α [44]. This class of histone demethylases removes methyl-group from histone H3 tails, leading to the loss of repressive histone marks. In detail, JMJD1A catalyses the formation of mono-methyl lysine 9 in H3 histone tail (H3K9me1) from di-methyl lysine (H3K9me2), whereas JMJD2B removes a methyl group from tri-methyl lysine 9 in H3 histone tail (H3K9me3) producing H2K9me2. The removal of these repressive histone marks has been associated with the restoration of the expression of self-renewal genes, such as *OCT-4* in SCs [44], as supported also by other authors that found JMJD1A and JMJD2C enhancing the expression of self-renewal genes in embryonic SCs [45].

In hypoxic condition SCs are prone to assume a phenotype more similar to ESCs, with enhanced capacity of differentiation and proliferation, as supported by several studies, that show hypoxia promoting de-differentiation of early committed ESCs reacquiring pluripotency [46], or hypoxic condition accelerating the process of reprogramming of iPSCs [47]. These observations underline that the epigenetic machinery in adult SCs is devoted not only to drive their differentiation but also to maintain their stemness [48-50], two opposite effects requiring a well-orchestrated control at the level of master genes driving cell differentiation and division.

Besides local changes in the chromatin structure in the region of HIF-target genes leading to their activation, hypoxia has also been found to provoke a dramatic decrease of gene transcription that seems to be influenced by epigenetic modifications [38,42]. Among the transcriptional repressive modifications hypoxia is also known to induce global deacetylation of histones [51], as well as increased H3K9me level induced by the up-regulation of histone methyltransferase G9a [52]. Increased level of global DNA methylation following the upregulation of the DNA methyltransferases (DNMTs) has also been reported in several studies [51].

An additional level of regulation controlled by hypoxia in SC niches are microRNA (miRs), short non-coding RNA molecules regulating, in a sequence-specific manner, gene expression via translational repression or mRNA degradation [53]. Under hypoxic conditions, miR-210 expression is significantly increased, modulating the levels of iron-sulphur cluster protein (ISCU), a protein which is involved in the mitochondrial electron transport chain [51]. Other groups of miRs seem to regulate vascular endothelial growth factor (VEGF) which, in turn, stimulates angiogenesis [54]. Interestingly, some of these miRs are downstream effectors of HIFs, providing further evidence that most changes induced by hypoxia are strictly under the control of these transcription factors. HIF-1α is also involved in cell cycle regulation, as shown in HSCs where heterozygous deletion of von Hippel-Lindau factor (VHL) causes enhanced HIF-1α expression and cell quiescence [55]. Moreover, in the hypoxic niche the proliferating hematopoietic cell fraction re-enters cell cycle quiescence. The HIF-1α target factors that potentially correlate with these effects of hypoxia in HSCs are VEGF and Cripto/GRP78 signaling, whose presence has been shown in the niche of various SCs, including HSCs an MSCs.

#### Role of hypoxia in the cancer SC niche

Most of the above-mentioned adaptations of adult SCs to hypoxia under healthy conditions have also been found in cancer stem cells (CSCs). These cells, which share many characteristics with normal SCs, seem to be necessary for tumor maintenance, progression, and malignancy [56]. As for adult SCs, most CSCs reside in hypoxic niches where their functions depend on several autocrine/paracrine factors, ECM molecules, and non-tumor cells. Notably, in respect to bulk tumor cells, the expression of HIF-1α is higher in CSCs leading to increase survival and progression to more aggressive and undifferentiated phenotypes [57]. Other findings have shown that oxygen levels are subjected to significant fluctuations in tumor niches and that these intermittent episodes of hypoxia and reoxygention are even more effective in promoting CSC survival and progression than continuous hypoxia [58].

All these observations clearly demonstrate that both normal and cancer stem cells develop several mechanisms of adaptation to hypoxia that provide them increased resistance to different stresses. This suggests useful cues to simulate under ex-vivo conditions a similar environment to improve the performances of SCs addressed to cell therapy applications.

### Hypoxia-dependent conditions improving ex-vivo SC survival

#### Hypoxic preconditioning of SCs

The poor vascularization of the injured tissues, especially if damaged by an ischemic insult, meets only partially the metabolic needs of the transplanted cells, hence more than 80-90% of cells undergo apoptosis within the first days after grafting [59]. In the attempt to confer more resistance to SCs, some strategies have been suggested to increase the cell ability to survive to hypoxia (Table 1).

**Table 1:** Effects of reduced oxygen tension on SC survival

**Cell type**	**pO**_**2**_**modulation**	**Increased cell survival/ mechanism**	**Host tissue/effects**	**Ref.**
Mouse peripheral blood mononuclear cells	Continuous hypoxia	Increased oxidative stress resistance	Ischemic hind limb/increased angiogenesis	[65]
Rat MSCs	Continuous hypoxia	Reduced apoptosis/activation of Akt, ERK, NADPH oxidase		[66]
BM-MSCs	Continuous hypoxia	Akt activation and cMET upregulation	Ischemic hind limb/increased angiogenesis	[67]
Skeletal myoblasts	Cyclic hypoxia	Improved resistance to lethal anoxia		[68]
Human MSCs	Continuous hypoxia		Infarcted swine heart/ increased cardiac contractility and angiogenesis	[69]
BM-MSCs	Anoxia	Reduced apoptosis	Diabetic cardiomyopathy/reduced remodeling	[70]
Murine MSCs	Continuous hypoxia	Increased Wnt4 expression	Ischemic hind limb/increased angiogenesis and MSC retention, proliferation, migration	[71]
Mouse BM-MSCs	Continuous hypoxia	HIF-1α-dependent CXCR4 and CXCR7 overexpression	Ischemic and reperfused kidney/ increased MSC survival, recruitment, proliferation	[72,82]
MSCs	CoCl_2_	HIF-1α-dependent CXCR4 overexpression	Acute kidney ischemia/ increased MSC retention, migration	[73]
HUVECs	Cyclic hypoxia	Increased cicloxygenase-2		[74]
MSCs	Cyclic hypoxia	HIF-1α dependent activation of Akt and miR-210 and CAP8AP2 upregulation	Infarcted heart/higher MSC survival	[76]
MSCs	Transgenic induction of miR-210	Increased CASP8AP2 expression	Infarcted heart/improved MSC grafting and cardiomyocyte protection	[77]
Endothelial cells	Continuous hypoxia	Reduced apoptosis/HIF-1α dependent miR-210 upregulation and receptor tyrosine kinase ligand Ephrin-A3 inhibition		[78]
MSCs	Cyclic hypoxia	HIF-1α-dependent Akt and miR-107 activation and PDC10 inhibition	Infarcted heart/increased MSC survival	[80]
MSCs	diazoxide	NF-kB activation		[88]
Skeletal myoblasts (SMs)	diazoxide	Prevented apoptosis/activation of Akt, bFGF, HGF, cycloxigenase-2	Infarcted rat heart/higher SM survival and increased cardiac contractility, angiogenesis	[89]

Since IP seems to be the most potent strategy to protect organs against severe ischemia, this approach was adopted in some clinical context, for instance to reduce the cardiac injury that follows coronary stent or bypass applications [19,20]. Almost conclusive findings demonstrate that mitochondria are the main intracellular orchestrator of IP-induced cytoprotection, following the opening of mitochondrial potassium channels (mitoKATP) that trigger anti-apoptotic responses [60]. Mitochondria primed by IP also contribute to cell survival by attenuating proton leakage from the inner membrane [61].

Taking advantages from the knowledge of the molecular mechanisms leading to cell survival during organ IP, in-vitro simulations of this strategy have been adopted to improve resistance of cultured cells against harmful conditions of hypoxia. Therefore “Hypoxia Preconditioning” (HP), which is characterized by intermittent periods of hypoxia, or anoxia, followed by reoxygenation [62-64], has been applied with different protocols to improve survival of cultured SCs.

Continuous hypoxia, rather than cyclic hypoxia, was also able to activate intracellular signal transduction pathways of survival in adult SCs, together with other processes useful for regenerative medicine, such as SC proliferation and paracrine activity [65-67]. However, the effectiveness of cycling/intermittent hypoxia/anoxia followed by reoxygenation was demonstrated to be superior than continuous hypoxia. Heider and Ashraf observed that cycles of intermittent anoxia and reoxygenation better preconditioned skeletal myoblasts than one-time continuous exposure to anoxia of the same duration and intensity [68]. Moreover, the leakage of lactate dehydrogenase from myoblasts subjected to lethal anoxia treatment was inversely related to the number of cycles used to prime cells, as well as other indexes of cell damage, including TUNEL positivity, hypercontracture and spherical morphology of cells. Nevertheless, most protective protocols have used a single long-term exposure to low oxygen tensions, equal or lower than 5%; hence, hereafter we will extend the term HP to non-lethal continuous hypoxia.

MSCs preconditioned with HP not only survived better to transplantation in a swine model of chronic myocardial ischemia but also ameliorated cardiac function [69]. Improved anti-apoptotic and anti-remodeling potency of bone marrow MSCs was also observed in a model of diabetic cardiomyopathy [70]. HP was demonstrated to be effective for the regeneration of other tissues besides the infarcted myocardium, including skeletal muscle fibers [71] and kidney [72]. Renal lesions due to acute ischemia were also regenerated using MSCs pretreated with cobalt chloride (CoCl_2_) which simulates HP, since divalent cations such as the Co^2+^ compete with iron and inhibit the activity of PHDs, leading to stabilization of HIF-1α [73]. Under this condition the effectiveness of cell therapy was enhanced by the activation of MSC migration towards the injured region of the kidney. Another evidence of the beneficial effects of HP in increasing cell survival was demonstrated by Daneau et al. [74] in human umbilical vein endothelial cells (HUVECs) that were subjected to three periods of one hour each at 0.5% O_2_ concentration, interrupted by 30 minutes of reoxygenation. These positive effects did not occur when three hours of continuous hypoxia were applied rather than the same time-period of intermittent hypoxia.

#### HIF-1α-dependent signal transduction pathway in SCs

As mentioned above, the efficacy of HP is mainly ascribed to HIF-1α activity. HIF-1α-dependent Akt phosphorylation was responsible of the increased survival in MSCs preconditioned with two cycles of 30 minutes of anoxia/reoxygenation [68]. Moreover, a selected group of miRs downstream to HIF-1α were found to protect MSCs against hypoxia [75]. Kim et al. [76] demonstrated that HP could increase the survival of MSCs by upregulating miR-210 and its concentration correlated to the number of anoxia/reoxygenation cycles. The principal target gene of miR-210 that is responsible of cytoprotection is FLASH/caspase-8-associated protein 2 (CAP8AP2), a molecule that significantly reduced apoptosis in the MSCs treated with HP when subjected to lethal anoxia. Accordingly, transgenic induction of miR-210 in MSCs promoted their survival as well and increased the resistance to death in MSC engrafted in the infarcted heart [77]. Strikingly, when miR-210-transduced MSCs were cultured with cardiomyocytes, some miRs were transferred to cardiac cells that acquired higher protection. Functional links between HIF-1α and miR-210 against apoptosis were also observed in hypoxic endothelial cells [78] and cancer cells [79]. Besides miR-210, HP was also able to induce miR-107 in MSCs as a consequence of HIF-1α activation [80]. One major putative target of miR-107 was identified as the programmed cell death-10 (PDC10) protein which is regulated by HIF-1α independently from CASP82.

Other targets of HIF-1α have been discovered in MSCs treated with HP, such as the SDF-1 receptors CXCR4 and CXCR7 [81,82]. Therefore, the effects of SDF-1 are potentiated in MSCs after HP as demonstrated in the ischemic and reperfused kidney [72]. The improvement of HIF-1α-dependent survival of HUVECs exposed to HP was correlated to an increased expression of cycloxygenase-2 [74]. Other mechanisms have been linked to the protective effects of HP, including Wnt-4 [71] and Notch-stimulated Jagged2 activation as observed in cultured CSCs [83,84].

#### Diazoxide treatment simulating IP

Pharmacological manipulations simulating the effects of HP in adult SCs would represent a more convenient alternative to hypoxic treatments due to the ease of drug administration. In this regard, MSCs were preconditioned with diazoxide, a mitoKATP channel opener extensively experienced as IP mimetic for cardiac protection [85,86]. The effectiveness of diazoxide in preventing cardiomyocytes apoptosis was correlated to multiple transduction signaling cascades involving a preliminary translocation of both Akt and PKCδ into mitochondria and the subsequent phosphorylation of mitoKATP channels, leading eventually to the inhibition of cytochrome c release into cytosol [87]. Other mechanisms related to the diazoxide treatment have been described, such as improved MSC survival through the activation of nuclear factor kB (NF-kB) [88] and increased protection and angiogenic properties in skeletal myoblasts via release of cytokines and growth factors [89]. Among them, VEGF exerts a dual role in promoting both tissue neovascularization [90] and cell survival [91,92], as also demonstrated in SCs [93,94]. Accordingly, VEGF can facilitate the commitment of circulating mononuclear cells towards the formation of EPCs [95,96] and their recruitment in ischemic tissues [97]. In addition, VEGF stimulates iPSCs to differentiate into cardiac muscle cells [98].

Besides diazoxide, several chemical compounds have also been demonstrated to be effective in preconditioning SCs by improving their survival, proliferation, and differentiation either in vitro or after transplantation in pre-clinical models (Table 2) [99-108]. Since the mechanisms of action of these drugs are different from those attributable to the hypoxic treatments, they will be not discussed further.

**Table 2:** Chemicals and cytokines useful for preconditioning SCs before their transplantation

**SC type**	**Chemical/cytokine**
Neural SCs	isoflurane [99], interleukin-6 [100]
EPCs	sevoflurane [101]
MSCs	oxytocin [102], angiopoietin-1 [103], hydrogen peroxide [104], transforming growth factor-α [105], , trimetazidine [106]
ADSCs	sydenafil [107]
Cardiac SCs	cobalt protoporphyrin [108]

### Effects of hypoxia pretreatments on SC differentiation

#### Hypoxic stimulation of neurogenesis

As described above, SC quiescence can be favored by the hypoxic environment of the niche. By contrast, other studies underline that hypoxia can be also responsible, at least in part, of the following steps in the life of SCs, namely proliferation and differentiation. Since quiescence, expansion, and differentiation are processes that cannot temporarily co-exist in the same cell, it is not clear how a condition of hypoxia can provoke such different biological events. A possible explanation is provided by the knowledge that hypoxia is not the only featuring factor operating in the SC niche because also different cell types and ECM/paracrine-related signals are present in that microenvironment. Thus, only a tunable orchestration of all components of the niche can allow SCs to take one or another direction towards a specific biological process. For example, studies focusing on adult neurogenesis underlined the relevant role played by the brain niche where neural stem cells (NSCs) reside and generate new neurons [109,110]. In the healthy human adult brain, the subventricular zone (SVZ) and specific areas of the hippocampus have been identified as regions where thousands of new neurons can stem from NSCs [111,112]. Since the physiological concentrations of oxygen in the brain range from 0.5% (midbrain) to 8% (pia) [113], it can be hypothesized that NSCs display intermediate levels of hypoxia during their migration from the niche towards other regions and consequently modulate their state of differentiation. Likewise, it has been suggested that an ischemic event in the brain can stimulate neurogenesis not only in the NSC niches but also in other injured areas where NSCs are migrated.

The involvement of brain hypoxia in promoting NSC proliferation and differentiation has encouraged the researches to pilot the NSC behavior under culture conditions of hypoxia. As extensively reviewed by Vieira et al. [114], several ex-vivo approaches have demonstrated that SC proliferation and differentiation into the neural lineage are enhanced in the presence of 2% to 5% O_2_ or using CoCl_2_ treatments. Besides adult NSCs and neural precursor cells, cobalt increased also MSC commitment towards dopaminergic neuron-like cells. Notably, the process of differentiation was associated with an increased expression of HIF-1α together with its target genes erythropoietin, VEGF, and p21 [115] (Table 3).

**Table 3:** Effects of reduced oxygen tension on SC differentiation

**Cell type**	**pO**_**2**_**modulation**	**Cell differentiation/ mechanism**	**Ref.**
BM-MSCs	CoCl_2_	Dopaminergic neuron-like cells/HIF-1α dependent activation of EPO, VEGF, p21	[115]
Rat MSCs	Continuous hypoxia	Increased chondrogenesis/activation of HIF-1α, Akt, p38 MAPK, SOX-9	[119]
Human MSCs	Continuous hypoxia	Inhibited osteogenesis/ HIF-1α dependent inhibition of RUNX2	[121]
MSCs	Continuous hypoxia	Inhibited hypertrophic chondrogenesis	[122]
BM-MSCs	Continuous hypoxia in alginate beads	Increased chondrogenesis/ HIF-1α dependent stimulation of SOX-9	[123]
Human BM-MSCs	Continuous hypoxia in gelatine hydrogel	Increased chondrogenesis	[124]
Human ADSCc	Continuous hypoxia and chondrogenic medium	Increased chondrogenesis	[126]
ADSCc	Continuous 2% oxygen tension	Decreased chondrogenesis and osteogenesis	[127]

The possible role of reactive oxygen species (ROS) in these models of neurogenesis has been highlighted, since oxidative stress can increase in SCs subjected to low oxygen concentrations [29,116]. Interestingly, the contribution of ROS was confirmed by studies in which neural differentiation was obtained in the PC12 cell line subjected to hyperoxia and prevented by antioxidant treatments [117]. However, although HIF-1α itself and several genes regulating cell redox response are known to be targeted by ROS [118], the mechanistic link which induces SCs to undergo differentiation remains to be elucidated.

#### Hypoxic stimulation of chondrogenesis

Cartilage is another tissue which is widely investigated for regenerative therapy and that is naturally subjected to low oxygen tension since it is devoid of any vasculature. Due to this particular characteristic, oxygen levels gradually decrease from both superficial and calcified zone to the inner zone of cartilage. There is a general agreement in stating that hypoxia can stimulate MSCs to differentiate into chondrocytes. Hypoxia enhanced chondro-specific differentiation of the MSC line C3H10T1/2 increasing the biosynthesis of both collagen type II and aggrecan by the p38 MAPK pathway [119]. Hypoxic conditions increased SOX-9 expression in human MSCs via HIF-1α but also HIF-2α seems to be involved, at least in articular chondrocyte differentiation [120]. On the contrary, hypoxia inhibits RUNX2 expression because of the upregulation of HIF-1α and TWIST [121]. Therefore, under hypoxic conditions MSCs are almost impeded to differentiate into osteoblasts, an event that instead is observed during the progression of osteoarthritis due to the vascularization of cartilage. Likewise, hypoxia inhibits collagen type X production counteracting hypertrophic chondrogenesis [122].

In view of regenerative medicine applications, Duval et al. [123] grew bone marrow MSCs (BM-MSCs) in alginate beads under hypoxic conditions without addition of exogenous growth factors. BM-MSCs underwent chondrogenesis after seven days, as confirmed by HIF-1α and SOX-9 overexpression. In another study similar results were obtained using gelatin-based hydrogel as substrate [124].

Stem cells with mesenchymal features like BM-MSCs that can be isolated from the adipose tissue (ADSCs) [125] showed also the potential to differentiate into chondrocytes when subjected to hypoxia [126]. Strikingly, O_2_ concentration as low as 2% decreased chondrogenesis and osteogenesis in ADSCs exposed to the corresponding differentiating media [127]. Therefore, oxygen tensions ranging from 2% to 5% should be considered as the optimal O_2_ concentrations for ADSC commitment towards chondrogenesis.

### Other putative mechanisms by which preconditioned SCs can improve organ function

Besides the enhanced survival and differentiation of the SCs themselves, it is possible that the hypoxic pretreatments of SCs produce other beneficial effects in the injured host tissue. It is known that several effects of SC therapy are due to the paracrine activity of these cells. Multipotent SCs synthesize a broad spectrum of soluble mediators that include molecules with immunomodulatory, anti-apoptotic, pro-angiogenic, and chemoattractive effects. For example, the transplantation of MSCs in the infarcted heart has been described to improve cardiac contractility especially because of the sustained release of growth factors from MSCs rather than their transdifferentiation into cardiac muscle cells [128]. However, partial oxygen pressures can significantly affect the profile of these paracrine mediators, and therefore, change the biological response of the target cells. In particular, several angiogenic growth factors that are normally released by MSCs, including VEGF and HGF, are produced to a greater extent if MSCs are exposed to hypoxic conditions [129]. Anyway, at least as a consequence of the enhanced viability of the preconditioned SCs in the injured tissue, the amount of released growth factors and their permanence during the regenerative process should be increased as well. Finally, it should be also taken into consideration that several approaches of tissue engineering require 3D scaffolds where oxygen diffusion to the embedded cells can be made difficult due to the thickness and low porosity of the supporting material [130]. Therefore, a preventive adaptation of SCs to a condition of partial hypoxia should improve the effectiveness of construct transplantation.

## Conclusions

Making SC transplantation a really efficient procedure is one of the major challenge for regenerative medicine. Discovering simple and safe strategies for increasing survival and favoring differentiation of grafted cells will pave the way for new suitable interventions for tissue repair. Treating SCs with HP have been demonstrated to increase resistance against cell death and to promote proliferation and differentiation towards specific cell lineages. However, easier pharmacological cell handling recapitulating decisive intracellular effects induced by HP or IP could represent a forward tool to improve cell therapy and encourage tissue engineering applications. Different factors such as autacoids (e.g. adenosine, bradykinin, opioids), cytokines (e.g. erythropoietin), their receptors and signal transduction pathways, and mitochondrial function are implicated in the protection induced by IP [131]. Several molecular steps are activated, including extracellular regulated kinase 1/2, phosphatidylinositol 3 kinase/Akt, protein kinase C, and protein kinase G, which are able to inhibit glycogen synthase kinase-3*β* and, in turn, the opening of the mitochondrial permeability transition pore [132]. Also mitochondrial ROS are consistently involved in the causative mechanisms of IP [133]. The protection induced by IP is not more operating after a few hours after the last cycle of ischemia/reperfusion but reappears 24 hours later. This “delayed” IP is due to the synthesis of protective proteins, including heat shock proteins, manganese superoxide dismutase, and inducible nitric oxide synthase [134].

According to this cascade of signaling events, a variety of drugs affecting these pathways could be investigated as IP mimetics to exert protection. For example, clinical trials have demonstrated that adenosine or erythropoietin administration are effective in cardioprotection by significantly reducing the infarct size [135,136]. Therefore, besides diazoxide, other drugs simulating hypoxic treatments should be investigated to provide protection to SCs before their transplantation.

Although low oxygen tension-based pretreatments have demonstrated to give considerable results on SC viability and functional performance, the in-vivo short-term permanence of these beneficial effects still represents a problem to be solved. In order to obtain a more stable action of SCs in the damaged tissues, gene therapy inducing the SC expression of adaptive molecules to the ischemic environment could likely represent a future approach. Alternatively, biomaterials releasing HP/IP mimetics and contemporary conveying SCs [137] could also prolong cell activity after grafting and improve the overall process of tissue regeneration. In addition, bioreactors for high-throughput cell yield providing hypoxic cell cultures chambers should be employed to give a sufficient amount of preconditioned SCs to regenerate the injured region [138,139]. Future interdisciplinary studies investigating these issues will surely contribute to upgrade these basic researches for clinical applications.

## Competing interests

The authors declare no competing interests in relation to this manuscript.

## Authors’ contributions

CM, EG, FB, MG, and CG designed the concept and collected information. CM wrote the manuscript. AP rearranged the section referring to the epigenetic changes induced by hypoxia upon resubmission. All authors read and approved the final manuscript.
